# The role of brown adipose tissue in mediating healthful longevity

**DOI:** 10.20517/jca.2024.01

**Published:** 2024-04-27

**Authors:** Jie Zhang, Berhanu Geresu Kibret, Dorothy E. Vatner, Stephen F. Vatner

**Affiliations:** 1 Department of Cell Biology and Molecular Medicine, Rutgers, New Jersey Medical School, Newark, NJ 07103, USA.; 2 Department of Medicine, Rutgers, New Jersey Medical School, Newark, NJ 07103, USA.

**Keywords:** Brown adipose tissue, healthful longevity, exercise, obesity, glucose and insulin protection, regulator of G protein signaling 14

## Abstract

There are two major subtypes of adipose tissue, i.e., white adipose tissue (WAT) and brown adipose tissue (BAT). It has been known for a long time that WAT mediates obesity and impairs healthful longevity. More recently, interest has focused on BAT, which, unlike WAT, actually augments healthful aging. The goal of this review is to examine the role of BAT in mediating healthful longevity. A major role for BAT and its related beige adipose tissue is thermogenesis, as a mechanism to maintain body temperature by producing heat through uncoupling protein 1 (UCP1) or through UCP1-independent thermogenic pathways. Our hypothesis is that healthful longevity is, in part, mediated by BAT. BAT protects against the major causes of impaired healthful longevity, i.e., obesity, diabetes, cardiovascular disorders, cancer, Alzheimer’s disease, reduced exercise tolerance, and impaired blood flow. Several genetically engineered mouse models have shown that BAT enhances healthful aging and that their BAT is more potent than wild-type (WT) BAT. For example, when BAT, which increases longevity and exercise performance in mice with disruption of the regulator of G protein signaling 14 (RGS14), is transplanted to WT mice, their exercise capacity is enhanced at 3 days after BAT transplantation, whereas BAT transplantation from WT to WT mice also resulted in increased exercise performance, but only at 8 weeks after transplantation. In view of the ability of BAT to mediate healthful longevity, it is likely that a pharmaceutical analog of BAT will become a novel therapeutic modality.

## Graphical Abstract

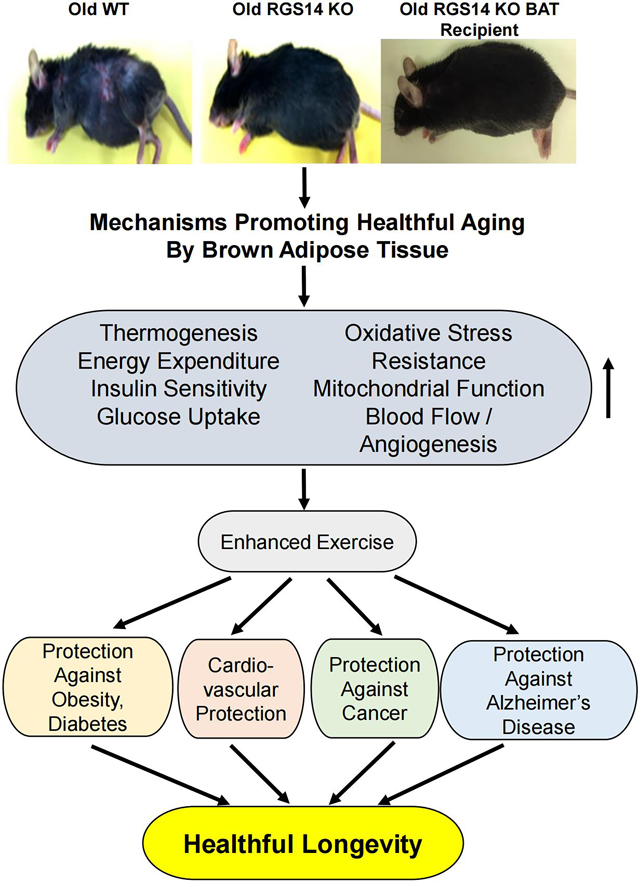

## INTRODUCTION

The goal of this review is to examine the role of brown adipose tissue (BAT) in mediating healthful longevity. This topic is of increasing importance as lifespan continues to increase, but is associated with adverse effects of diseases of aging, that not only decrease lifespan, but more importantly, decrease healthful lifespan. Our hypothesis is that the increased healthful lifespan is due in part to BAT. Much of the data supporting this hypothesis are derived from studying mouse models of healthful aging, a key feature of the current review.

However, in the past few years, the increase in longevity and healthful longevity has not been sustained and life expectancy has actually fallen in the U.S., which was triggered by an unprecedented rise in mortality associated with the COVID-19 pandemic, opioid overdose epidemic, and suicide^[1]^. According to a recent CDC report, life expectancy for the total U.S. population declined from 78.8 years in 2019 to 77.3 years in 2020, then further declined to 76.1 years in 2021, and then bounced back to 77.5 years in 2022^[2]^. This increase from 2022 does not fully offset the loss of 2.4 years of life expectancy between 2019 and 2021 that mostly resulted from increases in excess deaths due to the COVID-19 pandemic, opioid overdoses, and suicide^[1]^. For example, the number of drug overdose deaths increased by more than 16% from 2020 to 2021. From 2020 to 2021, opioid-involved death rates increased by over 15% and synthetic opioid-involved death rates (excluding methadone) increased by over 22%^[3]^. The current lifespan of 77.5 years is considerably longer than the lifespan prior to the year 2000. It has roughly doubled since 1860 and increased by a third since 1940^[4]^.

### White adipose tissue *vs.* brown adipose tissue

There are two major subtypes of adipose tissue, i.e., white adipose tissue (WAT) and brown adipose tissue (BAT). Whereas WAT plays a role in increasing obesity and impairing healthful longevity, BAT has the opposite effect. In addition to WAT and BAT, beige adipocytes are present in WAT and have functions similar to those of both WAT and BAT. It has been recognized for a long time that a major role for BAT and its related beige adipose tissue is thermogenesis as a mechanism to maintain body temperature by producing heat through uncoupling protein 1 (UCP1), which dissociates oxidative phosphorylation from ATP production, resulting in the release of heat^[5–7]^. Changes in BAT with aging reduce its effects on thermogenesis^[8]^. In addition, UCP1-independent thermogenic pathways have been found in BAT, beige adipocytes, and muscles^[9]^. These pathways include (1) creatine-substrate cycling in thermogenic adipocytes^[10–12]^; (2) Sacro-endoplasmic reticulum ATPase (SERCA)/Sarcolipin uncouple ATP hydrolysis from SERCA Ca^2+^ transport in BAT and muscle^[13–16]^; and (3) SERCA2b-mediated Ca^2+^ cycling in beige adipocytes^[17]^. BAT functions as a metabolic sink by oxidizing glucose and lipids, which produces heat. This has resulted in interest in BAT being a therapeutic modality to protect against obesity and diabetes. More recently, BAT has been shown to mediate longevity, but more importantly, healthful longevity.

Adipose tissue plays a vital role in regulating energy, and its activity relies on hormonal and nutritional signals that determine whether fat cells store excess nutrients as intracellular lipids or release stored energy as heat^[18]^. Changes associated with aging can affect the normal physiology of adipose tissues and alter their modulatory activity on energy regulation^[19]^.

The major WAT depots in the body are found in the visceral cavity (vWAT) and subcutaneous cavity (scWAT). Compared with BAT and Beige adipocytes, WAT has less fatty acid oxidation, mitochondria, respiratory chain, and peroxisome proliferator-activated receptor gamma coactivator-1 alpha (PGC-1α) levels^[20]^. Redistribution of WAT mass with advancing age is displayed by increased visceral adipose tissue in trunk and abdomen and reduced subcutaneous adipose tissue from limbs^[21,22]^. With aging, adipose tissue is characterized by dysregulated immune cells, preadipocytes, and senescent cells^[21,22]^.

BAT consists of small, multilocular adipocytes (each cell has many small lipid droplets) and is responsible for dissipating energy through uncoupled respiration to produce heat^[19,23]^. Brown adipocytes are smaller in size than white adipocytes, with lipid droplets surrounding the nucleus. Brown adipocytes have mitochondria dispersed between the droplets, which give these cells a brown appearance. The cytoplasm also contains Golgi apparatus and only a small amount of ribosomes and endoplasmic reticulum. BAT is mainly located in the interscapular space of mice, and in humans, it is found in the interscapular, supraclavicular, suprarenal, and para-aortic spaces^[24–27]^. Browning of WAT is referred to as “beiging” with the cells obtaining a brown fat-like morphology and function^[28]^.

Beige adipocytes are a distinct type of brown-like thermogenic adipocytes with multilocular morphology. They exist mainly in subcutaneous fat, but a small portion can also be found in visceral fat. Beige cells are generated through WAT browning, resulting in augmented non-shivering thermogenesis and metabolic capacity^[29,30]^. Beiging occurs predominantly in scWAT^[31]^. This process involves the upregulation of UCP1, a molecule that uncouples the respiratory chain from ATP synthesis, producing heat^[32,33]^. β-3 adrenergic receptors (β-3 ARs) mediate WAT browning during cold exposure^[29,34–36]^. Although β-1 ARs are expressed in BAT, they are not usually coupled to major functions in mature brown adipocytes^[37]^. β-1 ARs were reported to mediate preadipocyte recruitment, instead of WAT browning^[29]^. Although β-2 ARs mRNA and protein can be detected in BAT, they are most likely localized to the vascular system^[38,39]^. A recent report shows that β-2 ARs can activate human BAT^[40]^. In contrast to cold exposure, studies on local hyperthermia also revealed WAT browning^[41–43]^. Physical exercise copes with increased levels of specific molecules, including β -aminoisobutyric acid and irisin, which induce adipose tissue browning^[44,45]^. Calorie restriction and intermittent fasting also increase WAT browning and metabolic efficiency^[46–48]^, and the intestinal microbiome regulates WAT browning, as it modulates bile acid levels, which are important for thermogenic activation^[49]^. Beige adipocytes are functionally related to brown adipocytes, which dissipate energy in the form of heat. The induction of beige adipocytes in human WAT depots is postulated to improve glucose, lipid metabolism, and obesity. In addition to thermogenesis and energy expenditure, like BAT, beige adipose tissue is also associated with improved glucose and lipid homeostasis and improved insulin sensitivity^[50,51]^. Specific cellular mechanisms have been identified in BAT, which regulate BAT’s role in metabolism, e.g., disruption of IL-6 diminishes BAT’s role in glucose homeostasis and insulin protection^[52]^ and adipose-specific ablation of desnutrin/ATGL reduces BAT’s action by converting it to a WAT-like tissue^[53]^. A recent prospective clinical trial observed that in pre-diabetic humans, GLP1 analog treatment acutely induces IL-6 production by monocytes and IL-6 in the systemic circulation^[54]^. The same study showed that metronomic treatment with a GLP1 analog, liraglutide, leads to thermogenic adipose tissue activation in mice^[54]^.

### Secreting factors derived from BAT - brown adipokines or batokines

BAT and beige adipocytes have been identified as having a secretory role by releasing multiple autocrine and paracrine factors, to control the expansion and activity of BAT and the extent of WAT browning^[55,56]^. Additionally, endocrine factors derived from BAT can target peripheral tissues, such as WAT, liver, heart, pancreas, skeletal muscle, and bone. These factors derived from BAT are called brown adipokines or batokines^[55,56]^. Several batokines and their roles are identified, such as fibroblast growth factor 21 (FGF21)^[57–60]^, Interleukin 6 (IL-6)^[52,61]^, neuregulin 4 (NRG4)^[62]^, insulin-like growth factor 1 (IGF-1)^[63,64]^, CXCL14^[65]^, 12,13-diHOME^[66–68]^, Myostatin^[69]^, GDF-15^[70]^, and microRNAs (e.g., miR-99b)^[71]^.

FGF21 was reported to regulate glucose uptake in BAT and browning of WAT^[58]^, blood pressure regulation^[59]^, and hypertensive cardiac remodeling^[60]^. IL-6 was reported to be associated with metabolic improvements^[52]^ and hepatic gluconeogenesis enhancement^[61]^. A recent review of NRG4 has summarized its role in the regulation of energy homeostasis and glucolipid metabolism^[62]^. BAT-released IGF was reported to normalize glucose levels and reverse diabetes symptoms in a type 1 diabetes model^[63]^. Both CXCL14^[65]^ and GDF-15^[70]^ were reported to have effects on macrophages. 12,13-diHOME is associated with cardiac function^[66]^, fatty-acid transportation^[67]^, and increased fatty-acid uptake in skeletal muscle induced by exercise^[68]^. Elevated myostatin is associated with reduced exercise capacity, which was observed in the BAT-specific interferon regulator factor-4 (IRF4) KO mice^[72]^. In addition, miR-99b was reported to regulate hepatic production of FGF21^[71]^.

### BAT in humans from young to old

In infants, there is a high prevalence of BAT, but adults have less BAT, which is localized in a specific region of the body. Aherne and Hull state that in newborns, “many smaller masses of brown adipose tissue are present around the muscles and blood vessels of the neck. The main mass follows the course of the internal jugular vein and common carotid artery”^[73]^.

In some studies, BMI and body fat percentage had a negative association with BAT prevalence, while resting metabolic rate had a strong positive correlation^[26,74,75]^. Cypess *et al.* found the prevalence of detectable BAT was higher in women (7.5%) than in men (3.1%)^[25]^. Out of those with detectable BAT, 48.1% of individuals were less than 50 years old, 34% were 50–64 years old, and 17.9% were over 64 years old^[25]^. BAT activity was observed in 23 of the 24 subjects during cold exposure but not under thermoneutral conditions^[26]^. The activity was significantly lower in the overweight or obese subjects than in the lean subjects^[26]^. The prevalence of detectable BAT was 36% in women (9 out of 25 individuals) and 32% in men (10 out of 31 individuals) with 2-h cold exposure^[76]^. Cold-activated BAT was detected in 125 (48%) out of 260 subjects at a median age of 26 (20–72 years). Out of those with detectable BAT, 26% of females (20 out of 76) and 54% of males (100 out of 184) exhibited detectable BAT. Compared with subjects without detectable BAT, those with detectable BAT were younger and showed lower adiposity-related parameters such as BMI, body fat mass, and abdominal fat area^[77]^. The loss of BAT regeneration with aging promotes the development of obesity and metabolic disorders of aging^[78]^.

### BAT mediating longevity

Studies have indicated that aging reduces BAT activity, leading to thermal dysregulation and energy imbalance^[21,79,80]^. However, the effects of age on BAT mass have been inconsistent^[81–83]^. Some studies have reported that aging increases the amount of BAT^[81,82]^, whereas one study reported no change in BAT mass in rodents^[83]^. In addition, beige adipocyte formation declines with aging, which may be caused by changes in the adipose tissue microenvironment^[21,22]^.

**Figure 1.**
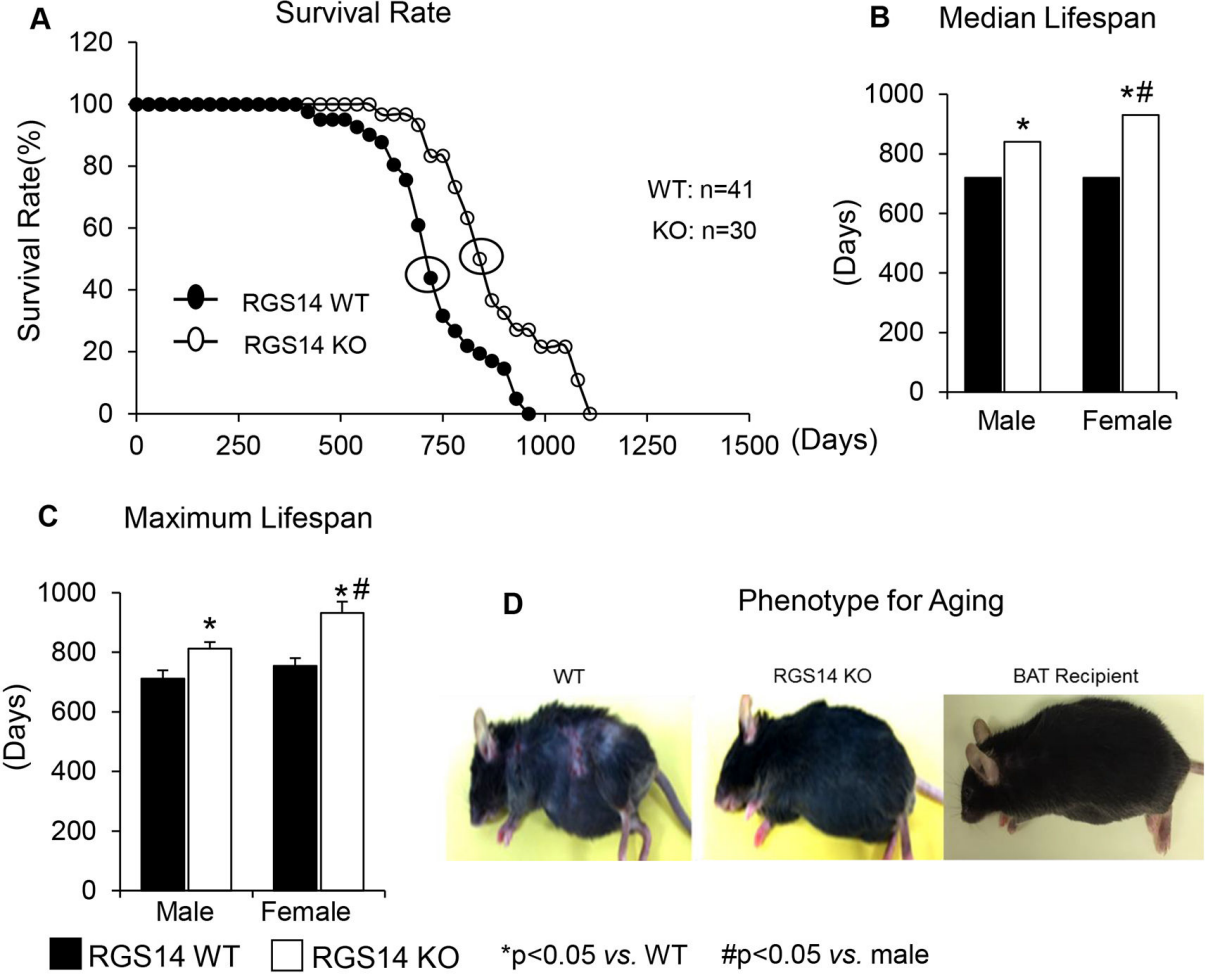
RGS14 KO Model of Longevity. (A) Kaplan-Meier survival curves for RGS14 KO and WT mice showed significantly augmented survival in RGS14 KO mice. (B and C) Median survival value and maximum lifespan were significantly greater in RGS14 KO mice than in WT mice for both males and females. In addition, medium and maximum lifespan were greater in female RGS14 KO mice than in male RGS14 KO mice. (D) Furthermore, 24-month-old RGS14 KO mice did not show the aging phenotype normally present in WT mice of similar age, including body atrophy, loss of hair, and greying of fur color. In support of the key role of BAT in aging, old WT RGS14 KO BAT recipient mice, which had BAT transplanted at 3–4 months of age, had the appearance of healthful aging similar to the old RGS14 KO mice. A representative example of each is shown in (D). For median lifespan analysis, Mood’s median test was used to determine differences in median lifespan. A Student’s t-test was used to test differences in maximum lifespan. Reprinted from Ref.^[23]^.

Several animal studies of aging have observed an association between BAT and aging, with increased age of median survival ranging from 13 to 68% compared to their wild type (WT) [Table 1], including Ames Dwarf mice^[84,85]^, Growth Hormone Receptor/Binding Protein (GHR/BP) knockout (KO) mice^[53,86]^, Phosphatase and Tensin Homolog transgenic (Pten^tg^) mice^[87]^, Regulator of G Protein Signaling 14 (RGS14) KO mice^[23]^, and Forkhead Box Protein A3 (Foxa3) KO mice^[88]^. Most of these studies have observed a correlation between aging and aspects of BAT, rather than a distinct examination of the extent to which surgical elimination of BAT affects lifespan. However, the model of disruption of the Regulator of G Protein Signaling 14 (RGS14), i.e., RGS14 KO, in mice is a model of extended longevity. When their BAT is transplanted to their WT, the RGS14 KO mouse without its BAT is no longer a model of longevity and the WT with the transplanted BAT becomes a model of longevity [Figure 1].

### BAT mediating healthful longevity

**Figure 2.**
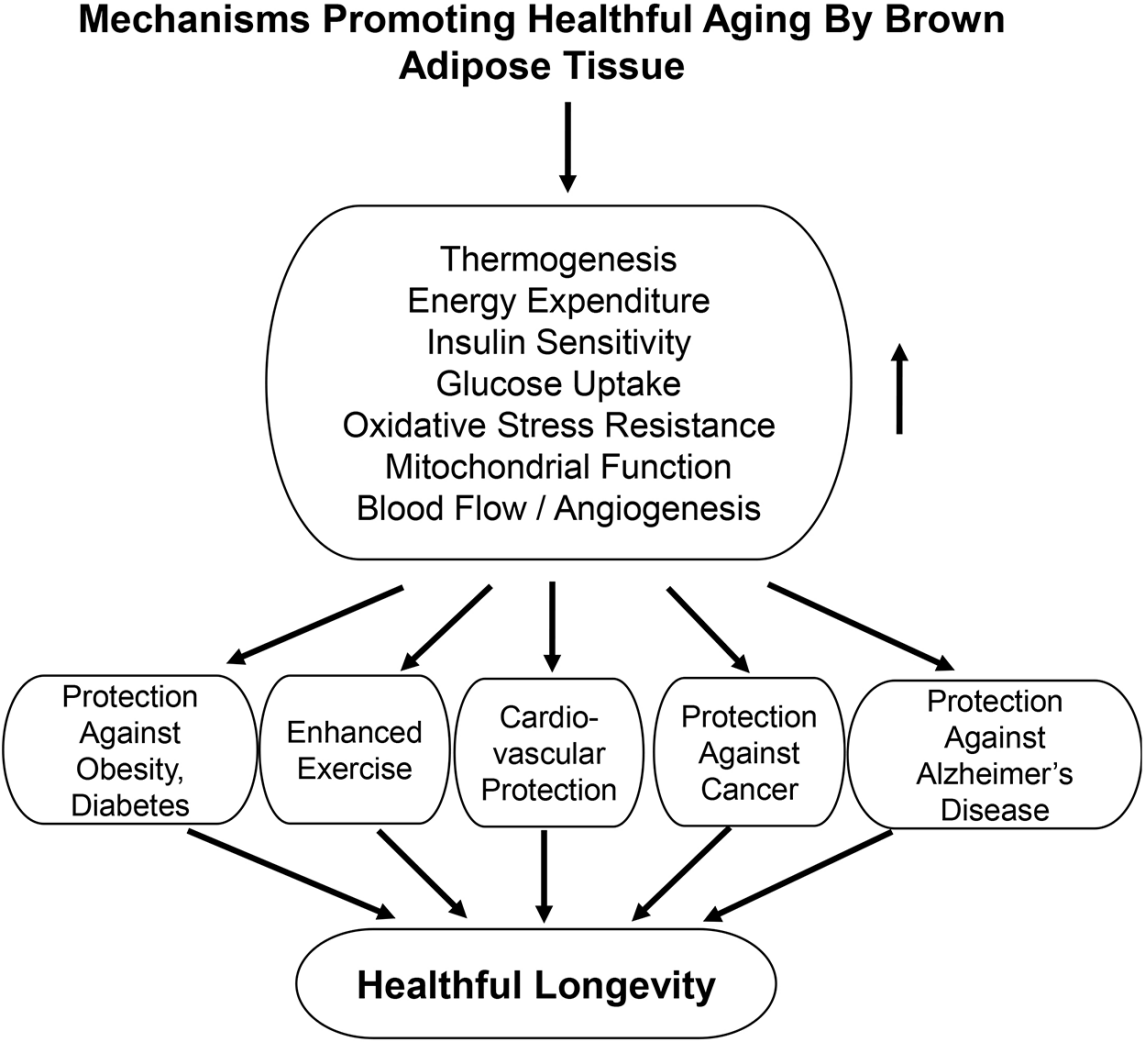
Mechanisms Promoting Healthful Aging By BAT. BAT leads to healthful aging by promoting thermogenesis, energy expenditure, insulin sensitivity, glucose uptake, oxidative stress resistance, mitochondrial function, and blood flow/angiogenesis. This results in protection against obesity, diabetes, exercise intolerance, cardiovascular disease, cancer, and Alzheimer’s disease.

More recently, interest has extended to BAT’s role in mediating healthful aging, primarily from data in genetically altered mouse models. Longevity mouse models with enhanced BAT activity / function are noted in Table 1. More importantly, these models mediate BAT’s role in healthful longevity, e.g., protection against obesity^[89,90]^, diabetes^[89,90]^, cardiovascular disorders^[91–93]^, cancer^[94–98]^, Alzheimer’s Disease^[99]^, stroke^[100,101]^, exercise intolerance^[72]^, and reduced blood flow^[72,102,103]^, all of which reduce healthful aging [Table 2]. Ames dwarf mice are protected against diabetes, CV stress, cancer, and Alzheimer’s Disease^[84,104–108]^. GHR/BP KO mice exhibit improved exercise capacity and are protected against obesity, hypertension, cancer, and Alzheimer’s Disease^[86,109–112]^. Pten^tg^ mice are protected against obesity and diabetes, cancer, and Alzheimer’s Disease^[87,113,114]^. Interestingly, whereas Pten TG is a model of healthful longevity, cardiac-specific Pten KO has also been reported to have cardiac stress protection^[115,116]^. RGS14 KO mice exhibit improved exercise capacity^[72]^, along with protection against obesity^[23]^ and glucose and insulin intolerance^[117]^, myocardial ischemia^[118]^, and hypertension^[119]^. Foxa3 KO is reported to protect against obesity and diabetes and cancer^[88,120]^ [Figure 2].

In addition, there are models with enhanced BAT function or extra BAT amount by BAT transplantation that exhibit aspects of healthful longevity. WT mice receiving BAT from another WT BAT mouse exhibit improved exercise capacity, as well as protection against obesity and diabetes and cancer^[52,72,121,122]^. BAT-specific p85αKO mice also protect against obesity and diabetes^[123]^. Adipose-Specific Neuregulin 4 Transgenic (Nrg4^tg^) mice protect against obesity and diabetes, cardiovascular stress, and cancer^[124–128]^. It has also been suggested that low levels of BAT in humans are associated with obesity and glucose intolerance, whereas those with higher BAT levels maintain lower body weights and more healthful aging^[129]^.

### BAT and obesity

By 2015, 108 million children and 604 million adults worldwide were obese, contributing to 2.4 million deaths globally^[130]^. The activation of BAT leads to increased energy expenditure through the uncoupling of mitochondrial respiration, generating heat, and utilizing glucose and fatty acids in the process, thereby protecting against obesity^[122,131]^. Thus, BAT plays a crucial role in averting obesity by serving as a metabolic regulator that actively disperses energy as heat. Unlike WAT, which stores surplus energy as fat, BAT distinguishes itself through its distinctive capacity for non-shivering thermogenesis^[132]^. The activation of BAT is enhanced in response to cold exposure, as sympathetic nervous system stimulation releases norepinephrine, promoting the thermogenic function of BAT^[133]^. BAT significantly expresses UCP1 and β3-adrenoceptors, which mediate the sympathetic drive to mobilize and upregulate UCP1 to promote a large amount of energy loss in the form of heat energy^[134]^. By burning calories to produce heat, BAT contributes significantly to overall energy expenditure, thereby preventing the accumulation of excess fat and mitigating the risk of obesity. Apart from cold-induced thermogenesis, there is a proposed role for BAT thermogenesis in mediating diet-induced thermogenesis. A high-fat diet is linked to an increase in thermogenic capacity, elevated BAT mass, and higher levels of UCP1. Conversely, ablation of UCP1 results in reduced thermogenic capacity and increased susceptibility to diet-induced obesity^[132]^, indicating an intricate interaction between mechanisms regulating energy balance and those controlling BAT. Recently, BAT transplantation has gained heightened attention in exploring the relationship between BAT and obesity. Studies have demonstrated that BAT transplantation enhances the utilization of stored energy, leading to a reduction in both body weight and body fat^[52,135]^. Beyond its role in energy metabolism, BAT emerges as a significant regulator of lipid metabolism, evidenced by a decrease in circulating triglycerides and an improvement in cholesterol profiles^[52,135]^. Overall, BAT serves as a dynamic metabolic force, actively burning calories to generate heat, thereby playing a crucial role in protecting against obesity by facilitating energy expenditure and metabolic well-being. Strategies to harness and activate BAT tissue hold promise for the development of anti-obesity interventions.

### BAT and diabetes

As noted in the previous section, due to its role in increasing energy expenditure and promoting glucose and fatty acid uptake^[122,131]^, BAT is recognized as an important tissue to combat the development of glucose intolerance and insulin resistance^[122,131]^ and protect against the pre-diabetic state^[77]^, obesity and allied metabolic disorders^[86]^. *Pten*^*tg*^ mice, a longevity mouse model with increased BAT activity, demonstrated increases in energy expenditure and improved glucose homeostasis^[87]^. The RGS14 KO mice also show protection against glucose intolerance and insulin resistance^[117]^. A recent human study reported that reduced BAT mass is associated with an increased incidence of type 2 diabetes and cardiovascular disease^[91]^.

Additionally, studies showed that increasing BAT mass by transplantation improves glucose metabolism and insulin sensitivity in mice^[52,136–138]^. BAT transplantation has been shown to protect against both type 1 diabetes by improving glycemia with increased IGF-1^[63,139]^ and type 2 diabetes by improving glucose tolerance with increased IL-6^[52]^ or adiponectin^[135]^. Ames dwarf mice, another aging model, also reported increased BAT activity along with greater oxygen consumption and energy expenditure^[84,140]^, whereas surgical removal of BAT in this model resulted in a decrease in insulin sensitivity^[84]^.

### BAT and cardiovascular disorders

The role of BAT in protecting against cardiovascular diseases is a major component of its ability to induce healthful aging. Several potential mechanisms mediate the protective effects of BAT on the cardiovascular system. As noted in the BAT and Obesity section, the activation of BAT leads to increased energy expenditure through the uncoupling of mitochondrial respiration, generating heat, and utilizing glucose and fatty acids in the process. BAT activation has also been associated with improved insulin sensitivity, reduced triglyceride levels, and favorable changes in lipid profiles, all of which contribute to cardiovascular protection^[52,141]^.

#### (1) BAT and Myocardial Ischemia

BAT has garnered significant attention in recent years due to its role in preventing myocardial ischemia, offering a novel perspective on cardiovascular health^[91–93]^. One study indicated that transplantation of CD29^+^ BAT-derived cells into the infarct border zone of acute myocardial infarction in rats resulted in reduced infarction area and improved left ventricular function^[142]^. The authors suggested that BAT-derived cells are useful for a new strategy in cardiomyocyte regeneration. Another study with BAT-derived stem cells also demonstrated smaller infarct size in rats with myocardial ischemia after 4 weeks of permanent coronary artery occlusion, with increased vessel density in the peri-infarct zone^[143]^. Recent studies also demonstrated that BAT dysfunction is associated with increased left ventricular mass and larger myocardial infarct size^[122,144]^, which also supports the role of BAT in protecting against myocardial ischemia. Similarly, the RGS14 KO mice have been shown to demonstrate myocardial ischemic protection^[118]^, with an important mechanism of that model’s ability to increase blood flow by angiogenesis^[72]^. A human study found that greater BAT activity was associated with reduced myocardial ischemia and protection against cardiac arrest and myocardial infarction^[145]^. Further exploration of BAT’s role in ischemic protection may open new avenues for innovative preventive and therapeutic strategies in the treatment of cardiovascular diseases.

#### (2) BAT and Heart Failure

Several reports have linked BAT and heart failure. In a murine model of heart failure with preserved ejection fraction (HFpEF), BAT function was reduced^[146]^. Tahara *et al.* reported the results of a 23-year-old female patient with heart failure who had low body temperature and suggested insufficient BAT-induced thermogenesis in this patient^[147]^. A more recent study reported that thoracic aortic constriction-induced heart failure reduced the thermogenic capacity of BAT in mice, leading to a significant reduction in body temperature with cold exposure^[148]^, while increased BAT function improved cardiac function in mice with thoracic aortic constriction^[148]^. Conversely, thoracic aortic constriction reduced systolic function in a mouse model of genetic BAT dysfunction, resulting in reduced survival after thoracic aortic constriction^[148]^. Similarly, another study suggested that BAT is activated in a model of catecholamine-induced cardiomyopathy, resulting in cardioprotection and protection against pathological left ventricle remodeling^[149]^.

#### (3) BAT and Hypertension

The prior literature on BAT and hypertension is controversial. One study showed that a transgenic model of reduced obesity and ablation of BAT (UCP - diphtheria toxin Achain (UCP-DTA)) mice^[150]^ is associated with systemic hypertension^[151]^. In contrast, another showed that an angiotensin type II agonist induces hypertension and enhances the browning of WAT^[152]^. Furthermore, research showed that adenosine A_2A_ receptor KO (A_2A_RKO) mouse is a hypertension model^[153]^ with interscapular BAT (iBAT) dysfunction^[60]^. The RGS14 KO mouse model is also protected against hypertension^[119]^, with an important mechanism involving its ability to increase blood flow through angiogenesis^[72]^. Additionally, perivascular fat and its browning have been reported to play a role in the development of hypertension^[154,155]^, including regulation of vascular contractility^[156]^ and vasodilation^[157]^. A recent study found that browning of perivascular adipose tissue prevents vascular dysfunction and reduces angiotensin II-induced hypertension in mice^[158]^. It has been suggested that BAT-secreted factors (batokines) contribute to the regulation of blood pressure^[159]^. For example, BAT secretes increased FGF21 after stimulation, and administration of FGF21 lowers blood pressure^[59]^. However, higher serum FGF21 levels are associated with higher blood pressure in humans^[160,161]^. Treatment with another batokine, IGF-1, lowers blood pressure by stimulating nitric oxide production from vascular endothelium and smooth muscle cells^[162,163]^. In addition, another study showed that BAT resulted in reduced vascular contractility through the Nox4-derived H_2_O_2_ pathway^[164]^.

In humans, thermogenic brown and beige adipose tissue are considered to have protective effects on the vasculature, as individuals with detectable thermogenic adipose tissue have reduced risk for hypertension and coronary artery disease, relative to individuals without thermogenic adipose tissue^[91]^.

### BAT and blood flow and angiogenesis

Compared to WAT, active BAT is highly vascularized with abundant mitochondria that produce heat through uncoupled respiration^[165]^. It has been shown that by stimulating angiogenesis and the conversion of WAT to brown-like adipocytes, weight gain in obese mice can be inhibited^[166]^. The thermogenesis function of BAT relies on blood flow to be supplied with nutrients and oxygen and for the distribution of the generated heat to the rest of the body. VEGF is an important angiogenic factor regulating angiogenesis, arteriogenesis, and blood flow. A direct connection between VEGF and BAT has already been established, as VEGF is known to play a direct and positive role in the activation of BAT^[167]^. VEGF also acts in an endocrine and paracrine manner in BAT by stimulating the proliferation of vascular endothelial cells^[168]^. Our recent study found that RGS14 KO mice have increased VEGF expression in the skeletal muscle and BAT^[72]^. Removing BAT from RGS14 KO mice resulted in the loss of a significant increase in hindlimb perfusion, while the addition of RGS14 KO BAT to WT mice led to increases in perfusion^[72]^. This addition also led to changes in the vasculature, with RGS14 KO BAT recipients exhibiting increases in capillary and arteriole density^[72]^. Other studies have shown that the activation of BAT is accompanied by the proliferation of blood vessels^[102]^, transplanted BAT from C57B/L6 mice becomes re-vascularized^[52]^, and receiving BAT from Fat-1 transgenic mice upregulates VEGF levels in endogenous BAT^[103]^. BAT transplantation effectively reverses skin sclerosis in mice through mechanisms involving inflammation reduction and promotion of angiogenesis^[169]^. In contrast, a recent study found the opposite, i.e., that the transplanted BAT derived from C57B/L6 mice did not improve blood flow or VEGF levels in HFD-fed mice^[170]^. Genetic deletion, as well as pharmacological inhibition of endothelial VEGFR1, increased adipose angiogenesis and browning of subcutaneous adipose tissue, leading to elevated thermogenesis^[171]^.

### BAT and stroke

Based on the role of BAT in energy metabolism, it has been suggested that the inhibition of BAT thermogenesis could facilitate the induction of therapeutic hypothermia for fever reduction or improve outcomes in stroke through a lowering of metabolic oxygen demand^[172]^. Some studies reported the potential role of BAT in a cerebral ischemic rat model. O’Shaughnessy *et al*. reported that BAT activity correlated with resting oxygen consumption in the cerebral ischemic group^[100]^. Another study found that hypobaric hypoxia preconditioning significantly attenuated the increases in cellular ischemia and injury indicators in the hypothalamus, along with reduced BAT weight^[101]^.

### BAT and exercise

Enhanced exercise capacity is not only a feature of healthful aging, but also is a therapy for aging patients and patients with cardiovascular disease. Exercise is a healthy way to reduce body weight by activating the sympathetic nervous system, accelerating the decomposition of fat, and promoting the utilization and consumption of energy in skeletal muscle^[173–175]^. During aging, it is known that progressive loss of exercise capacity relates to loss of skeletal muscle mass and tissue function^[176]^. Decreased muscle mitochondrial function contributes to the loss of skeletal muscle function during aging^[177–181]^. Regular exercise or exercise training protects against decreased muscle function during aging^[182,183]^, frailty status^[184,185]^, and neurodegeneration^[186,187]^.

Numerous studies have suggested that exercise may play a role in regulating BAT activation. Exercise boosts the expression of UCP1 and genes associated with mitochondria biogenesis, thereby improving BAT’s heat production capacity^[6]^. For instance, swim training in rodents over six to eight weeks increased UCP1 protein levels in BAT^[188,189]^. Similarly, treadmill exercise in rodents for 6–8 weeks increased BAT activity and cytochrome oxidase activity, oxygen consumption rates, and BAT-specific gene markers, e.g., UCP1, FGF21, and PGC1α^[190,191]^. However, conflicting findings also exist, with some studies suggesting that exercise may reduce the thermogenic effect of BAT. In rats, six to eight weeks of moderate-intensity treadmill exercise led to decreased UCP1 expression in BAT and a reduction in total BAT mass^[192,193]^. Human studies also showed inconsistent results regarding the role of exercise on BAT modulation, with some indicating that high-intensity physical activities can increase BAT density^[194]^, while others report that exercise decreases glucose uptake in BAT^[195–197]^. While most of these studies have shown that exercise increases BAT, relatively few have shown that BAT increases exercise performance.

**Figure 3.**
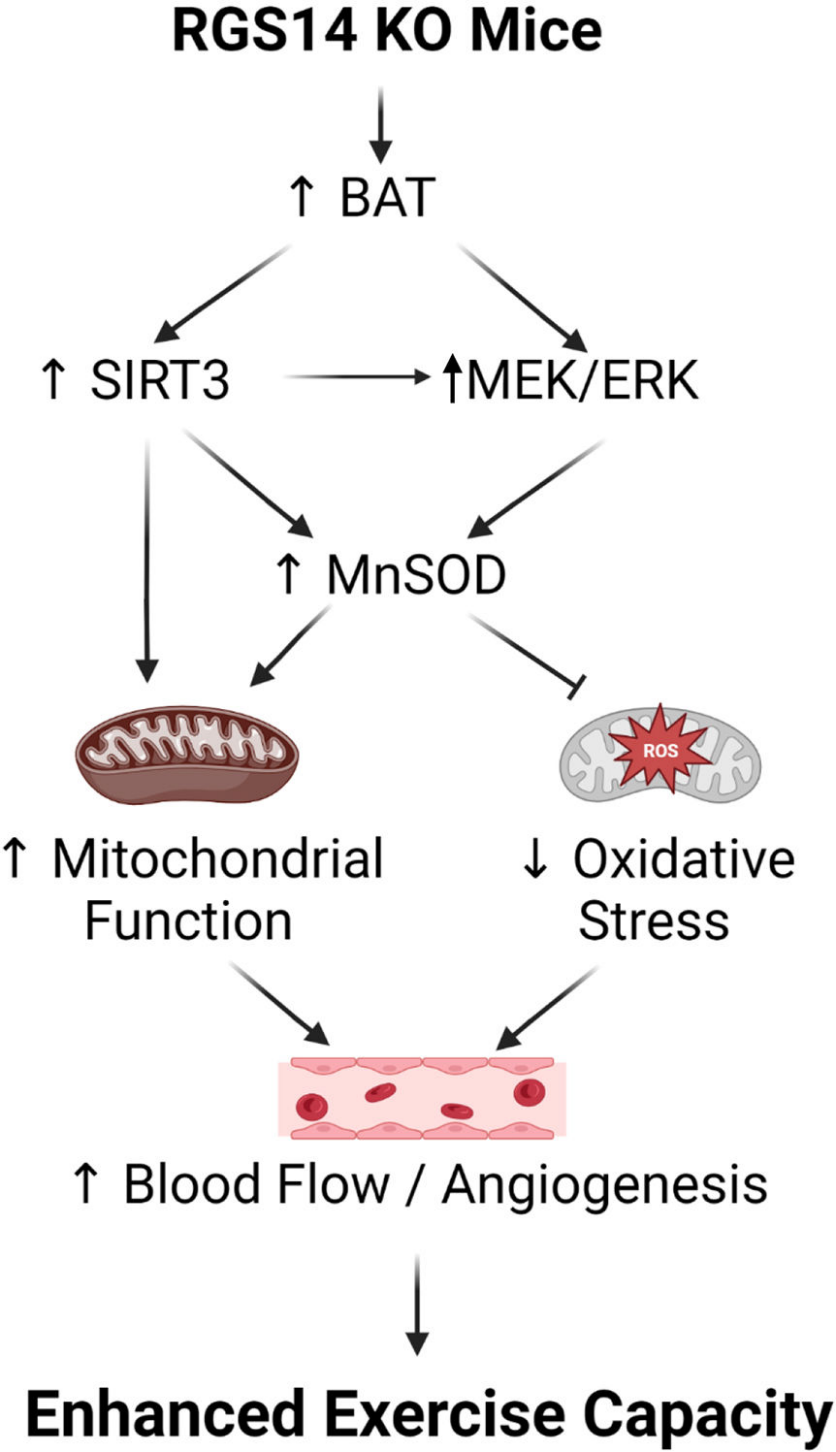
Mechanisms mediating enhanced exercise capacity in RGS14 KO and its uniquely powerful BAT. Multiple mechanisms mediated the enhanced exercise capacity in RGS14 KO mice. The most important mechanism is BAT, which mediates SIRT3, MnSOD, MEK/ERK, and VEGF pathways. These mechanisms regulate exercise capacity by improving mitochondrial function, providing protection against oxidative stress, and improving blood flow/angiogenesis. Reprinted from Ref.^[72]^.

**Figure 4.**
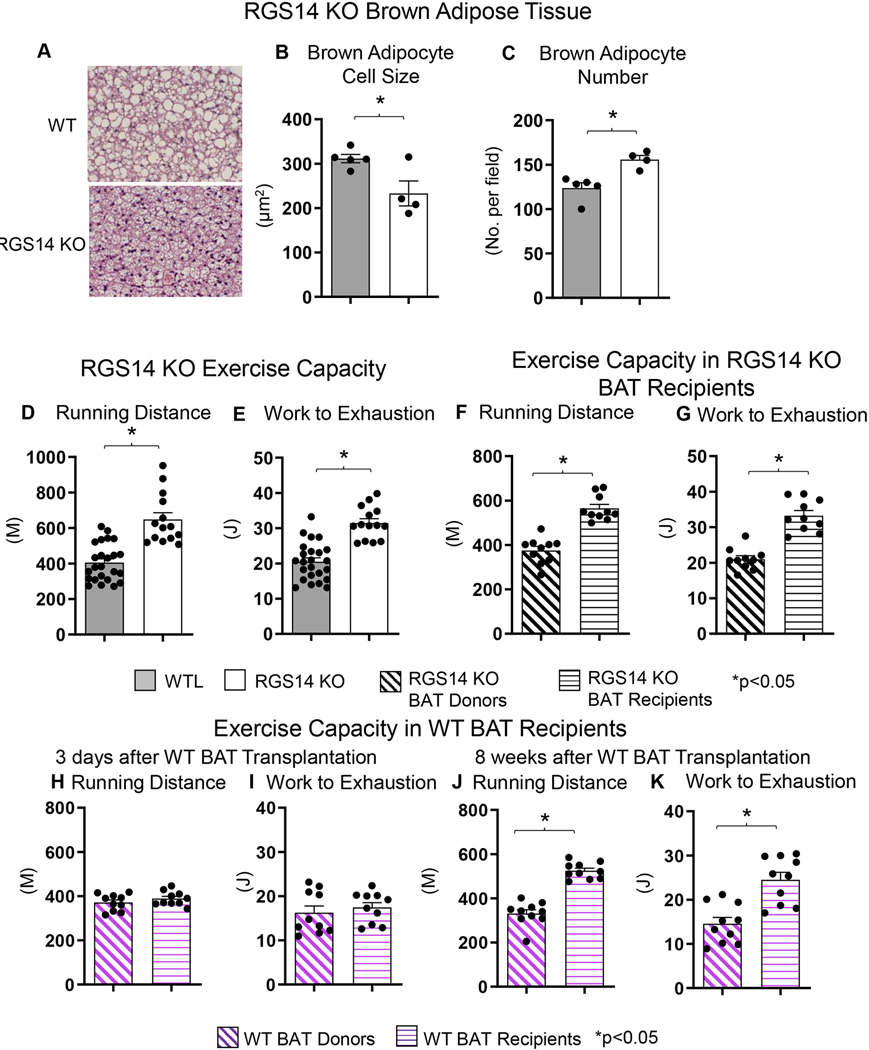
Increased BAT Cell Numbers and Increased Exercise Capacity in RGS14 KO Mice. RGS14 KO mice exhibited smaller brown adipocytes (A and B), and an increased number of brown adipocytes (A and C) than WT control mice. RGS14 KO mice ran longer distances (D) with increased work to exhaustion (E) compared to WT littermates. BAT transplantation from RGS14 KO mice to WT mice led to a reversal of phenotype, such that RGS14 KO BAT recipients exhibited improved running distance (F) and greater work to exhaustion (G) compared to RGS14 KO BAT donors, at 3 days after RGS14 KO BAT transplantation. In contrast, there was no improvement in running distance and work to exhaustion at 3 days after transplantation of BAT from C57BL6/J WT mice to other C57BL6/J WT mice (H and I). It required 8 weeks to achieve enhanced running distance and work to exhaustion in C57BI/6J WT mice with BAT transplantation from other C57BL6/J WT mice (J and K). Reprinted from Ref.^[72]^.

One example of a genetic model demonstrating that BAT can enhance exercise performance is that of RGS14 KO mice, a healthful lifespan model, mediated by increased BAT^[23]^. One mechanism mediating the increase in healthful lifespan is enhanced exercise capacity, a feature of the RGS14 KO mouse^[72]^ [Figure 3]. RGS14 KO mice demonstrated 160% ± 9% increased maximal running distance and 154% ± 6% increased work to exhaustion, compared to WT mice. Similarly, RGS14 KO BAT transplanted to WT mice demonstrated a 151% ± 5% increased maximal running distance and 158% ± 7% increased work to exhaustion, which corresponded to the enhanced exercise capacity of RGS14 KO mice. The enhanced exercise capacity observed in WT mice with RGS14 KO BAT transplants was observed at three days after BAT transplantation, whereas BAT transplantation from WT to WT mice also resulted in increased exercise performance, but not at 3 days, but only at 8 weeks after transplantation [Figure 4]^[72]^.

**Figure 5.**
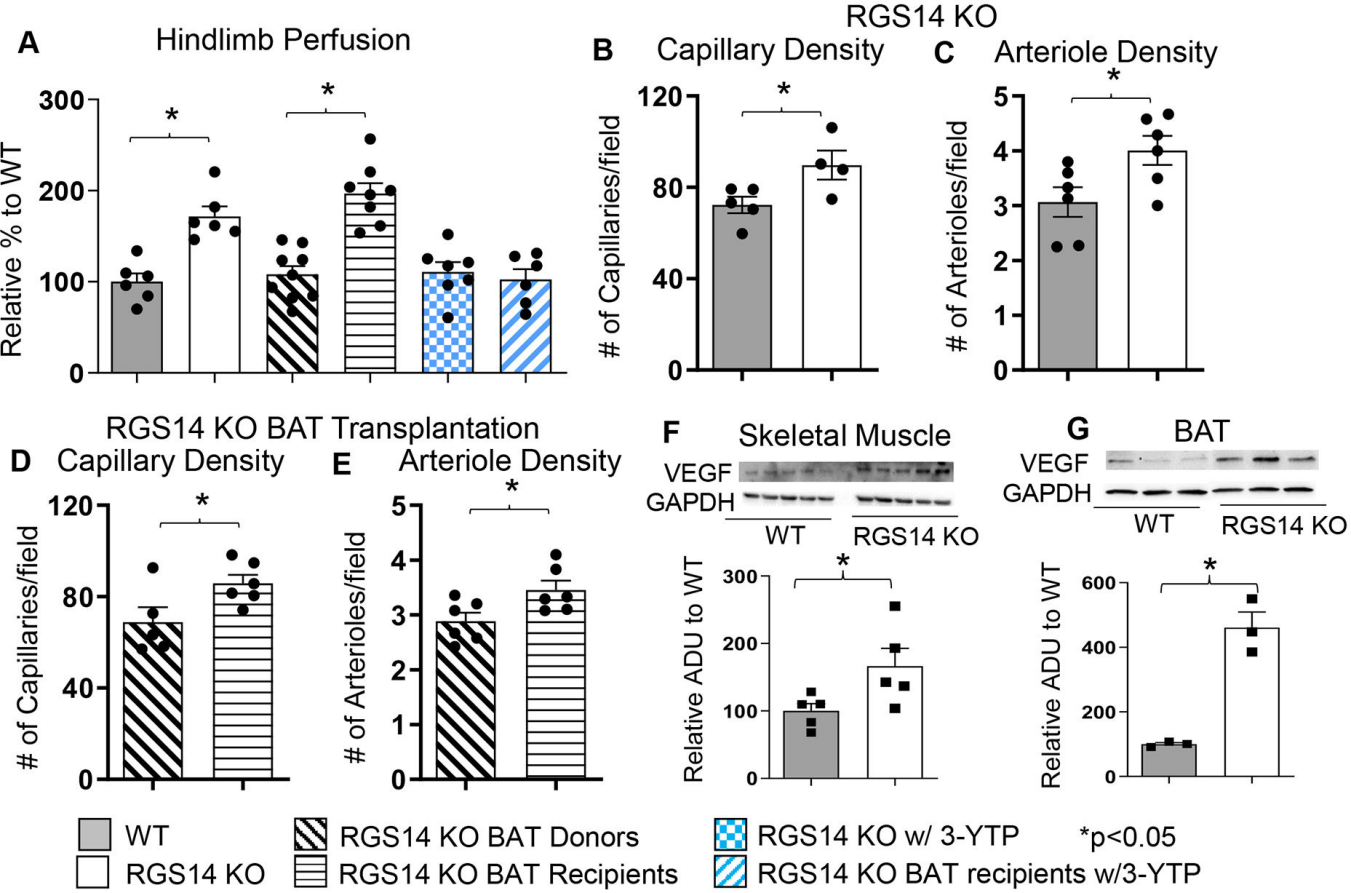
Enhanced Exercise by RGS14 KO Mice Is Mediated by BAT and Angiogenesis /Arteriogenesis Increasing HindLimb Blood Flow. Non-linear contrast imaging was used to measure hindlimb blood flow. The average data are presented as % of WT perfusion, which is represented as 100% (A). Hindlimb blood flow was higher in RGS14 KO mice compared to WT mice, and higher in WT mice that received RGS14 KO BAT, at 3 days after transplantation (A and B), while RGS14 KO BAT donors lost their enhanced hindlimb perfusion, with results similar to WT mice (A). With treatment of the SIRT3 inhibitor, 3-YTP, both RGS14 KO mice and RGS14 KO BAT recipients lost their enhanced hindlimb perfusion (A). Angiogenesis (reflected by capillary density) and arteriogenesis (reflected by arteriole density) were both increased in skeletal muscle of RGS14 KO mice (B and C) and RGS14 KO BAT recipients (D and E), which correlated with increased VEGF in skeletal muscle (F) and BAT (G). Reprinted from Ref.^[72]^.

The BAT-induced enhanced exercise capacity was mediated by (1) mitochondrial biogenesis and SIRT3; and (2) antioxidant defense and the MEK/ERK pathway, and increased hind limb perfusion [Figure 5]. Thus, BAT from WT or from RGS14 KO mice mediates enhanced exercise capacity, but the BAT from RGS14 KO mice was more powerful than from WT [Figure 4].

SIRT3, a mitochondrial sirtuin deacetylase, regulates the expression of many BAT mitochondrial proteins including UCP1^[198]^, and is also upregulated with exercise in animal models^[199]^. We have previously shown that the SIRT3 is upregulated in the BAT and skeletal muscle of RGS14 KO mice along with enhanced mitochondrial biogenesis^[72]^, and therefore, SIRT3 is considered an important regulator of exercise capacity in this model. These changes correlated with exercise capacity, such that RGS14 KO × SIRT3 KO mice did not show the enhanced exercise capacity of RGS14 KO mice^[72]^. The role of SIRT3 was necessary for the enhanced exercise capacity seen in the RGS14 KO mouse and WT mice with BAT transplantation, since the enhancement of exercise capacity upon RGS14 KO BAT transplantation to WT mice was not observed when BAT was transplanted from RGS14 KO × SIRT3 KO mice, even 6 months after transplantation^[72]^. MnSOD is a primary mitochondrial ROS scavenging enzyme, which can be activated by SIRT3^[200]^. SIRT3 and MnSOD (SOD2) have been linked to improved exercise, showing both that SIRT3 can improve exercise performance and conversely that exercise can lead to increased SIRT3^[199,201,202]^. Other studies have also shown that SIRT3 maintains BAT morphology and function and protects against obesity and age-related metabolic diseases^[203]^. The RGS14 KO mouse exhibits increased MnSOD activity, which also contributes to its enhanced exercise capacity, as confirmed by partial genetic ablation of MnSOD, which abolished the enhanced exercise capacity^[72]^.

RGS14 itself is also known to directly affect ERK signaling^[204]^, which is involved in angiogenesis/arteriogenesis^[205,206]^. The enhanced exercise capacity of RGS14 KO mice is also regulated by the MEK/ERK pathway, as this enhancement of exercise capacity was abolished by treatment with a MEK inhibitor, U0126^[72]^. Another powerful mediator of exercise performance is blood flow. RGS14 KO mice exhibit enhanced hindlimb blood flow, which is accompanied by increases in angiogenesis and arteriogenesis in the hindlimb vasculature, leading to increased capillary and arteriole density^[72]^ [Figure 5]. RGS14 KO mice have increased VEGF expression, a key angiogenic factor, in the skeletal muscle and BAT^[72]^. Moreover, VEGF plays a positive role in the activation and expansion of BAT^[167]^. VEGF also acts in an endocrine and paracrine manner in BAT by stimulating the proliferation of vascular endothelial cells^[168]^. Removing BAT from RGS14 KO mice resulted in the loss of a significant increase in hindlimb perfusion, while the addition of RGS14 KO BAT to WT mice led to increases in hind limb perfusion, along with RGS14 KO BAT recipients exhibiting increases in capillary and arteriole density^[72]^.

In contrast to the RGS14 KO data showing a positive action of BAT in mediating the enhanced exercise capacity, another study suggested that BAT from IRF4 KO mice might contribute negatively to skeletal muscle performance^[69]^. In that study, BAT from IRF4 KO mice produced and secreted myostatin^[72]^, which negatively regulates skeletal muscle cell differentiation^[69]^.

### BAT and cancer

Cancer is the leading cause of mortality in more than 100 countries worldwide^[207]^. The multifaceted involvement of BAT in the realm of cancer is becoming increasingly apparent. While BAT has conventionally been recognized for its contributions to thermogenesis and energy expenditure, it has now emerged as a factor influencing the development and progression of cancer. Most studies on the relationship between BAT and cancer have shown an increased prevalence of BAT activity in cancer patients or animals^[94–98]^. BAT mass or size was smaller in the cancer groups mainly due to the weight loss as a response to anorexia during the development of cachexia^[94]^. In addition, inhibition of WAT browning ameliorates the severity of cancer-associated cachexia with skin tumors^[208]^. Another study identified a significant association between the expression of UCP1 and improved overall survival in a cohort of patients with colorectal cancer^[209]^. Seki *et al.* observed significant tumor suppression in immunocompetent mice subcutaneously implanted with colorectal cancer cells, when the mice were housed in a 4 °C environment compared to those in 30 °C^[210]^. This environmental difference led to an upregulation of BAT and an impressive 80% reduction in tumor growth by day 20 post-tumor inoculation, underscoring the potential involvement of BAT in colorectal cancer progression^[210]^. An investigation into the connection between hepatocellular cancer and BAT in a mouse model demonstrated that the removal of BAT resulted in increased tumor growth^[211]^. This was accompanied by a more pronounced increase in liver weight and serum triacylglycerol levels.

However, not all studies have found that BAT protects against cancer. One study found no difference in the prevalence of activated BAT in cancer patients *vs.* that in healthy control subjects^[212]^. Other studies showed that BAT exerts an adverse effect on cancer^[96,213,214]^. In addition, an accelerated tumor growth rate has been shown with BAT or WAT^[213]^. BAT also plays a major role in breast cancer as well. One study revealed a high expression of markers associated with BAT and beige adipocytes in breast cancer xenografts, suggesting that thermal characteristics might play a pivotal role in the progression of breast cancer^[98]^. Consistent with this discovery, a retrospective analysis of data from 96 breast cancer patients who underwent FDG PET/CT scans for routine staging uncovered a three-fold higher BAT activity in breast cancer patients compared to controls with other types of cancers^[95]^. Cancer-associated cachexia, characterized by weakness, fat loss, and muscle wasting, is the primary contributor to complications in individuals with malignancies, resulting in diminished quality of life and unfavorable outcomes^[215,216]^. Investigations examining the role of BAT in cancer cachexia have yielded inconsistent findings. Some studies suggest that thermogenic fat plays a role in cancer cachexia due to its pivotal functions in heat production and energy balance^[217,218]^. Conversely, recent retrospective analyses of cancer patients propose that BAT is not associated with cancer-associated cachexia and does not exacerbate overall survival outcomes in individuals with cachexia^[219,220]^.

### BAT and Alzheimer’s disease

There is evidence that the age-associated thermoregulatory deficit induces diverse metabolic changes associated with Alzheimer’s Disease development. BAT has been involved in various functions that prevent Alzheimer’s Disease, such as regulating energy metabolism, secreting hormones, improving insulin sensitivity, and increasing glucose utilization^[99]^. BAT decreased significantly in an 18-month-old Alzheimer’s Disease mouse model, suggesting a potential role for BAT in protecting against Alzheimer’s disease^[221]^.

Characteristics of Alzheimer’s Disease are the accumulation of neurofibrillary tangles, amyloid plaques, neuropil threads, and dystrophic neurites containing hyperphosphorylated tau^[222–224]^. Hypothermia is one of the notable causes of tau hyperphosphorylation. Previous studies reveal that each degree Celsius below normothermic conditions induced an 80% rise in tau phosphorylation at the pThr212 and pSer396/pSer404 epitopes^[225,226]^. Decreased BAT function during aging may explain the thermoregulatory deficits in the elderly and the underlying mechanisms of Alzheimer’s Disease^[121]^.

Activation of BAT leads to an increased release of FGF21. FGF21, in turn, reduces brain oxidative stress and neuroinflammation by enhancing antioxidant activity and diminishing proinflammatory cytokines such as TNF-α and IL-6, critical factors in Alzheimer’s Disease pathogenesis^[227]^. Hormones produced by BAT, including adiponectin and leptin, contribute to neuroprotective effects by inhibiting proinflammatory cytokines and suppressing Aβ production, thereby attenuating tau phosphorylation^[228,229]^, providing additional evidence of BAT’s involvement in Alzheimer’s Disease.

Diabetes/insulin resistance is another risk factor for the incidence of Alzheimer’s Disease. Impaired brain insulin signaling can instigate neurocognitive diseases, and impaired glucose uptake is among the hallmark deficits in the Alzheimer’s Disease brain^[230,231]^. Taken together, these studies indicate that BAT could be a novel target in Alzheimer’s Disease therapy.

## CONCLUSIONS

It has been recognized for a long time that obesity mediated by WAT is a major cause of reducing both longevity and healthful longevity. More recently, there has been interest in another type of adipose tissue, BAT, with a mechanism of thermogenesis, which helps maintain body temperature. Most prior studies on BAT have focused on its ability to reduce obesity and protect against diabetes. More recently, the focus has shifted to a role for BAT in mediating other aspects of healthful longevity [Figure 2]. Several mouse models derived from genetic mutations have increased BAT and have been shown to mediate healthful longevity [Tables 1 and 2]. One of these newer models, i.e., RGS14 KO, has a BAT that is more powerful than BAT in WT controls and is involved in mediating the extension of healthful longevity, and not only protects against obesity, cardiovascular disease, glucose intolerance, and Alzheimer’s disease, but also exhibits improved exercise performance and angiogenesis. In view of the ability of BAT to mediate healthful longevity, it is likely that a pharmaceutical analog of BAT will become a novel therapeutic modality.

## Figures and Tables

**Table 1.** BAT models of longevity

Model	References	Sex	Age of median survival (Days)		
			Mutant	Control (WT)	% increase

Ames dwarf	[53]	M	1,076	723	49
		F	1,206	718	68
GHR/BP KO	[79]	M	975	629	55
		F	1,031	749	38
Pten^tg^	[55]	M	880	780	13
		F	915	796	16
RGS14KO	[14]	M	840	720	17
		F	930	720	29
Foxa3 KO	[56]	M	1,100	850	29

Ames dwarf: Prop1^df/df^; GHR/BP KO: growth hormone receptor/binding protein KO; Pten^tg^ : phosphatase and tensin homolog transgenic; RGS14 KO: regulator of G protein signaling 14 KO; Foxa3 KO: Forkhead box protein A3 KO. Median survival data of Foxa3 KOmice is extrapolated from graph in Yang *et al.*^[56]^.

**Table 2.** BAT models of healthy longevity

Mouse model	References	Aspects of healthful longevity					
		Improved Obesity exercise protection	CV stress/coronary protection	Hypertensio protection	Glucose, insulin n tolerance & diabetes protection	Cancer protection	Alzheimer’s disease protection

WT BAT transplantation	[39,70,91,92]	**	*		*		
BAT-specific p85α KO	[93]	*			*		
Nrg4 TG	[94–98]	*	*		*	*	
Ames dwarf	[52,73–77]		*		*	*	*
GHR/BP KO	[54,78–80]	**				*	*
Pten^tg^	[55,81,83]	*		*	*	*	*
RGS14KO	[14,70]	**	*	*	*		
Foxa3 KO	[56,90]	*			*	*	

* : Positive results; Nrg4 TG: adipose-specific neuregulin 4 transgenic; Ames dwarf: prop1^df/df^; GHR/BP KO: growth hormone receptor/binding protein KO; Pten^tg^: phosphatase and tensin homolog transgenic; RGS14 KO: regulator of G protein signaling 14 KO; Foxa3 KO: forkhead box protein A3 KO.

## Data Availability

Not applicable.
